# Rediscovering the Therapeutic Potential of Agarwood in the Management of Chronic Inflammatory Diseases

**DOI:** 10.3390/molecules27093038

**Published:** 2022-05-09

**Authors:** Juman Mohammed Rasmi Alamil, Keshav Raj Paudel, Yinghan Chan, Dikaia Xenaki, Jithendra Panneerselvam, Sachin Kumar Singh, Monica Gulati, Niraj Kumar Jha, Deepak Kumar, Parteek Prasher, Gaurav Gupta, Raniya Malik, Brian George Oliver, Philip Michael Hansbro, Kamal Dua, Dinesh Kumar Chellappan

**Affiliations:** 1 School of Postgraduate Studies, International Medical University (IMU), Kuala Lumpur 57000, Malaysia; 00000034715@student.imu.edu.my; 2 Centre of Inflammation, Centenary Institute and University of Technology Sydney, Faculty of Science, School of Life Sciences, Sydney, NSW 2007, Australia; KeshavRaj.Paudel@uts.edu.au (K.R.P.); Philip.Hansbro@uts.edu.au (P.M.H.); 3 School of Pharmacy, International Medical University (IMU), Kuala Lumpur 57000, Malaysia; YinghanChan@imu.edu.my; 4 Woolcock Institute of Medical Research, University of Sydney, Sydney, NSW 2006, Australia; dia.xenaki@sydney.edu.au (D.X.); Brian.Oliver@uts.edu.au (B.G.O.); 5 Department of Pharmaceutical Technology, School of Pharmacy, International Medical University (IMU), Kuala Lumpur 57000, Malaysia; Jithendra@imu.edu.my; 6 School of Pharmaceutical Sciences, Lovely Professional University, Phagwara 144411, India; sachin.16030@lpu.co.in (S.K.S.); hof.lfams@lpu.co.in (M.G.); 7 Faculty of Health, Australian Research Centre in Complementary and Integrative Medicine, University of Technology Sydney, Ultimo, NSW 2007, Australia; 8 Department of Biotechnology, School of Engineering & Technology (SET), Sharda University, Greater Noida 201310, India; niraj.jha@sharda.ac.in; 9 Department of Pharmaceutical Chemistry, School of Pharmaceutical Sciences, Shoolini University, Solan 173229, India; deepakkumar@shooliniuniversity.com; 10 Department of Chemistry, University of Petroleum & Energy Studies, Dehradun 248007, India; pprasher@ddn.upes.ac.in; 11 School of Pharmacy, Suresh Gyan Vihar University, Jagatpura, Jaipur 302017, India; drgaurav.gupta@mygyanvihar.com; 12 Department of Pharmacology, Saveetha Dental College, Saveetha Institute of Medical and Technical Sciences, Saveetha University, Chennai 602105, India; 13 Uttaranchal Institute of Pharmaceutical Sciences, Uttaranchal University, Dehradun 248007, India; 14 DeÁurora Pty Ltd., Dean, VIC 3363, Australia; raniya.malik@deaurora.com.au; 15 School of Life Sciences, Faculty of Science, University of Technology Sydney, Ultimo, NSW 2007, Australia; 16 Discipline of Pharmacy, Graduate School of Health, University of Technology Sydney, Ultimo, NSW 2007, Australia; 17 Department of Life Sciences, School of Pharmacy, International Medical University (IMU), Kuala Lumpur 57000, Malaysia

**Keywords:** herbs, agarwood, agarwood oil, inflammation, anti-inflammatory, therapeutics

## Abstract

The inflammatory response is a central aspect of the human immune system that acts as a defense mechanism to protect the body against infections and injuries. A dysregulated inflammatory response is a major health concern, as it can disrupt homeostasis and lead to a plethora of chronic inflammatory conditions. These chronic inflammatory diseases are one of the major causes of morbidity and mortality worldwide and the need for them to be managed in the long term has become a crucial task to alleviate symptoms and improve patients’ overall quality of life. Although various synthetic anti-inflammatory agents have been developed to date, these medications are associated with several adverse effects that have led to poor therapeutic outcomes. The hunt for novel alternatives to modulate underlying chronic inflammatory processes has unveiled nature to be a plentiful source. One such example is agarwood, which is a valuable resinous wood from the trees of *Aquilaria* spp. Agarwood has been widely utilized for medicinal purposes since ancient times due to its ability to relieve pain, asthmatic symptoms, and arrest vomiting. In terms of inflammation, the major constituent of agarwood, agarwood oil, has been shown to possess multiple bioactive compounds that can regulate molecular mechanisms of chronic inflammation, thereby producing a multitude of pharmacological functions for treating various inflammatory disorders. As such, agarwood oil presents great potential to be developed as a novel anti-inflammatory therapeutic to overcome the drawbacks of existing therapies and improve treatment outcomes. In this review, we have summarized the current literature on agarwood and its bioactive components and have highlighted the potential roles of agarwood oil in treating various chronic inflammatory diseases.

## 1. Introduction

Inflammation refers to an evolutionarily conserved process that involves the activation of both immune and non-immune cells [1]. It is characterized by a hallmark of signs and symptoms which may or may not be observed by the naked eye. These include redness, pain, swelling, heat, and loss of physiological function. The pathophysiological reasoning behind these signs and symptoms is explained by the complex processes that occur seconds to hours following exposure to causative factors that can be any external stimulus like pathogens, allergens, toxic materials, or foreign bodies, or it may be an internal stimulus due to some impairment in tissue functioning [2,3]. Ideally, such an inflammatory response can help defend the host from viruses, bacteria, toxins, and infections via the elimination of pathogens, thereby promoting tissue repair and healing [1]. As a result, the impending injury can be effectively minimized, thereby facilitating the restoration of tissue homeostasis, leading to the subsequent resolution of acute inflammatory processes. Nevertheless, if inflammation remains uncontrolled or unresolved, it may lead to chronic inflammatory responses that occur well beyond the presence of the causative stimuli [1,4]. In general, an inflammatory response is considered acute when it has an impromptu onset and lasts for no longer than a few days, whereas subacute inflammation is the one that lasts from 2 to 6 weeks. Chronic inflammation, on the other hand, has a gradual onset and lasts for prolonged periods of months to years, and it is usually associated with permanent damage to the affected site [5]. As such, chronic inflammation is considered the leading factor that contributes to the development of many diseases including different types of infections, atherosclerosis, autoimmune diseases, and malignancies. It also results in the progression of many aging illnesses [6]. Thus, given the huge socioeconomic and public health burden brought upon by chronic inflammatory diseases, the development of therapeutic agents to aid the resolution of inflammation is highly desirable.

Despite advancements in medical research and technologies, which had led to the development of various synthetic drugs for the treatment of chronic inflammatory diseases such as non-steroidal anti-inflammatory drugs (NSAIDs), corticosteroids, and immunosuppressants to treat these diseases, multiple recent studies have revealed drug-related toxicities, iatrogenic reactions, as well as adverse reactions that may affect the eventual therapeutic outcomes [7,8,9,10]. Hence, there is an impending need to address these issues through the discovery of novel anti-inflammatory therapies that can improve pharmacological response whilst minimizing adverse events. Over the years, there has been increasing interest in the use of natural products from medicinal and aromatic plants, functional foods, and their active constituents for the treatment of various diseases, as they possess an extended spectrum of pharmacological effects with improved toxicological profiles. Therefore, they offer the opportunity to elicit high therapeutic efficacy at the minimum effective dose with the least adverse reactions [11,12,13].

In terms of chronic inflammation, numerous studies have shown that plant-based therapeutics are effective in modulating the inflammatory mechanisms and mediators in the biological system to overcome various inflammation-related disorders [14,15,16]. Agarwood, a valuable resinous wood from the trees of *Aquilaria* spp., is an example of a plant that has exhibited tremendous potential for the treatment of various chronic inflammatory diseases [17,18]. Traditionally, agarwood has been widely utilized in aromatics, incense, religious, as well as medicinal preparations for centuries, in which medicinal application represents one of its most essential applications. For example, agarwood has been used to relieve high fever, cough, rheumatism, and has been used as a carminative medicine to treat gastric disorders in traditional Chinese medicine. It has also been utilized as a *qi*-regulating drug for relieving pain, arresting vomiting, and regulating respiration to relieve asthmatic symptoms. Agarwood is also commonly used in aromatherapy to treat neurodegenerative, digestive, and sedative diseases in traditional Arabian medicine [19,20]. Generally, agarwood oil is thought to be the main active constituent of agarwood, where modern research has shown that the beneficial pharmacological properties of agarwood oil, including its anti-inflammatory properties, can be attributed to the presence of a wide range of bioactive compounds, such as flavonoids, terpenoids, chromones, phenolic acids, steroids, and alkanes [20,21,22,23]. There has also been an increasing number of new compounds that are being identified and isolated from agarwood via phytochemical studies [19]. Recent innovative research has highlighted new compounds such as 2-(2-phenylethyl) chromone derivatives from agarwood that possess significant anti-inflammatory activity through inhibition of nitric oxide production from mice macrophage cells (RAW264.7) [24] and protection against acid-induced apoptosis of gastric epithelial cells [25]. To highlight the pharmacological potential of agarwood, we reviewed the current literature and collected recent information on the potential of agarwood from scientific search engines such as PubMed and Scopus. In this review, we offer a brief insight into various chronic inflammatory diseases and present the anti-inflammatory potential of agarwood oil and its bioactive compounds for treating various diseases, summarizing some of the most recent studies performed in this field of research.

## 2. Overview of Inflammation

**Figure 1:**
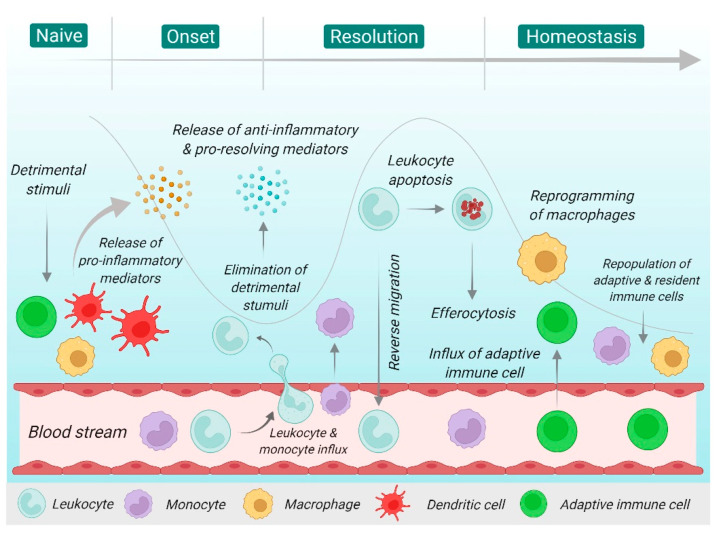
Cellular events during an acute inflammatory response.

Inflammation is typically characterized by a cascade of events that comprise an induction phase, which is followed by a peak phase of inflammation and subsequently the resolution phase [26,27] (Figure 1). The induction phase is initiated by the detection of endogenous and exogenous danger signals from biologically, chemically, or mechanically induced tissue damage [26]. An inflammatory environment modulates the signaling pathways that engage a network of innate and adaptive immune cells, as well as tissue components such as extracellular matrix, stromal fibroblasts, vascular networks, and soluble molecular messengers including cytokines, chemokines, and plasma proteins [26,28]. Although the processes of an inflammatory response are dependent on the exact nature of the initial stimulus and the site of damage, they share a common mechanistic pathway, namely, (i) recognition of detrimental stimuli by the cell surface pattern receptors; (ii) activation of inflammatory pathways; (iii) release of inflammatory markers; and (iv) recruitment of inflammatory cells [4,28]. Upon elimination of the detrimental stimuli and danger signal, the resolution phase is initiated, and it is crucial for the restoration of tissue homeostasis. However, prolonged, or intensified infiltration of various inflammatory cells may lead to chronic inflammation that could persist over months or years [10,26]. It is explained by the accumulation of lymphocytes, macrophages, and plasma cells where the inflammatory response is taking place. It is believed that the inflammatory mediators released by macrophages such as interleukins (IL)-1, -6, -13, -17, and tumor necrosis factor-α (TNF-α) are what trigger further reaction and push more cells like CD4+ and CD8+ to be recruited to the site of action and they, in turn, produce more mediators that sustain and amplify the inflammatory response [29]. Chronic inflammation has been implicated in various disease states including the development of autoimmunity, leading to excessive tissue damage, dysregulation of healing processes, and tissue fibrosis. Hence, timely resolution of acute inflammation is essential to avoid persistent chronic inflammation and the undesirable development of chronic inflammatory diseases [26,30].

During the onset of inflammation, the detrimental stimuli are detected by resident cells and the inflammatory cascade will be initiated via the active release of soluble pro-inflammatory mediators. This is followed by delayed monocyte emigration and the upregulation of adhesion molecules by circulating leucocytes and endothelial cells, leading to an influx of neutrophils and eosinophils, as well as macrophages from the bloodstream into the affected site. The leucocytes are responsible for the elimination of microorganisms and tissue debris via phagocytosis. The resolution phase is characterized by the removal of inflammatory infiltrate where the production, function, and signaling of pro-inflammatory cytokines are limited. The process is followed by neutrophil apoptosis and monocytes efferocytosis, which clears dysfunctional cells from the site of injury. The production of pro-resolving mediators is induced via the reprogramming of macrophages from classically to alternatively activated cells. At the same time, non-apoptotic cells leave the site of injury through reverse migration or lymphatic migration. Lastly, adaptive and resident immune cells repopulate the tissue, thereby resolving the acute inflammatory processes and returning to tissue homeostasis [26,27,31]. The summary of the inflammatory processes in different body organs is shown in Table 1.

## 3. Natural Products in Modern Drug Development

Natural products derived from medicinal plants, herbs, functional foods, as well as their active constituents have been widely investigated and are utilized for their potential in treating various human diseases since ancient times [15,54,55]. According to the World Health Organization (WHO), it is estimated that approximately 65% of the global population incorporates traditional medicine into therapeutic uses currently, on which ethnobotanical studies have greatly contributed to the discovery and identification of various plants with potent biological action [9]. The growing popularity of plant-based therapeutics can be attributed to their lower production costs in contrast to synthetic pharmaceuticals, as the costs of setting up and maintaining the production system for mass growing plants as well as the collection and curation of plant extracts are remarkably low. As such, the challenge of lack of access to medicines in rural areas and low-income countries can be overcome as plant-based therapeutics can be accessible by patients at lower and more affordable prices [54,56]. In addition, as they are naturally occurring, plant-based therapeutics have better toxicological profiles as compared to chemically synthesized compounds, which can improve safety, efficacy, and overall therapeutic outcomes in patients [8,10,57].

In terms of inflammatory diseases, traditional and herbal preparations have been used as anti-inflammatory therapies for thousands of years in addition to modern medicine, some of which had even been developed into western medicine drugs for their proven effectiveness and are being studied for the ideal way for them to be delivered to the targeted area [58]. *Curcuma longa*, for example, has proven effective in many inflammatory disorders, most importantly rheumatoid arthritis, gastric ulcers, irritable bowel syndrome, and inflammatory bowel disease. Another example is *Zingiber officinale*, which has been found to reduce the production of inflammatory mediators resulting in the reduction of the symptoms of disorders like osteoarthritis and rheumatoid arthritis [13]. *Eriobotrya japonica* leaf extract was found to combat airway inflammation in allergic asthma induced by ovalbumin in a mice model. The beneficial activity of the extract was due to the remarkable decrease in the level of nitric oxide, eosinophil peroxidase, IL-4, and IL-13 in the bronchoalveolar lavage fluid and IgE in serum [59]. The list goes on with many other herbs and plants that were proven for their effectiveness through modern research and studies, many of which have already approached their final stages in being developed as drugs to officially treat various inflammatory disorders. Agarwood is one example of a plant that has gained considerable attention for its medicinal properties, including its anti-inflammatory potential for treating various chronic inflammatory diseases. Apart from its anti-inflammatory properties, agarwood also possesses a wide range of biological actions that were observed and proven in various studies. These include neuronal activity in which it works as a sedative, anxiolytic, antidepressant, antimicrobial activity against *Staphylococcus aureus*, methicillin resistant staphylococcus aureus (MRSA), *Candida albicans*, and *Bacillus subtilis* possess anticancer, as well as analgesic, gastrointestinal regulation, and anti-diabetic activities [60,61]. Here, we will be specifically focusing on the anti-inflammatory potential of agarwood in treating various chronic inflammatory diseases, which will be discussed in the later sections of this review.

### 3.1. An Overview of Agarwood: Origin, Uses, and Distribution of Agarwood

Agarwood, also known as *chen xiang* in China, *jinkoh* in Japan, or *oud* in the Middle East, is a highly precious, aromatic, non-timber forest product of *Aquilaria* spp., and it has been a part of traditional Chinese medicine and Ayurvedic for centuries [62]. For example, in India and China, it is used as a medicine to treat digestive tract diseases such as loss of appetite, vomiting, and diarrhea, as well as respiratory diseases such as asthma and bronchitis as it has an effect to reduce cough, sleep disorders, and pain relief, etc. Apart from that, traditional and cultural uses of agarwood oil have long been known in festivals, religious ceremonies, and burned as a fancy perfuming oil while welcoming guests. In addition, many manufacturers are now using agarwood and agarwood oil to make different kinds of goods like personal care products including shampoo and soap, decorative sculptures, wooden boxes, and beads, as well as paper. Recently, the development of agarwood oil use in making fine, expensive fragrances has progressed tremendously [63,64,65]. Generally, the healthy wood of the trees of *Aquilaria* spp. is white and soft without the presence of scented resins. The formation of agarwood in a natural environment only occurs due to certain external factors, such as microbial invasion, animal grazing, insect attack, or lightning strike, leading to the secretion of resin as a defense reaction of the trees. The secreted resin will be deposited around the wounds or rotting parts of the trunk over several years, resulting in an accumulation of volatile compounds that would eventually form agarwood [66,67]. *Aquilaria* belongs to the Thymelaeaceae family of angiosperms, and there is a total of 31 *Aquilaria* species that have been documented worldwide. Among which, about 19 of them are recognized to be agarwood-producing with the rest of the species requiring further investigation to determine their competencies in producing agarwood. Some common examples of *Aquilaria* species that are reported to produce fragrant resin include *A. malaccensis, A. sinensis, A. rugosa, A. filaria, A. subintegra,* and *A. beccariana* [19,66,68]. These agarwood-producing species are often found in lowland tropical forests that are widely distributed in areas ranging from east India throughout Southeast Asia, as well as southern China. Specifically, Malaysia and Indonesia are the two major countries where agarwood originated [66]. In Table 2, we have summarized the distribution of various *Aquilaria* species based on existing documentation.

As mentioned, agarwood has extremely high demand worldwide as a raw material for aromatic food ingredients, perfumes, incense, religious purposes, and medicinal purposes. Most agarwood is traded in different forms of its derivatives, including solid wood pieces that are individually traded, wood chips, flakes, powder, and oil [62,72]. Depending on its quality, global prices of agarwood may range from 2000 to 10,000 USD per kilogram for the wood itself, or 6000 USD per kilogram for the wood chips. It is also very valuable due to the rareness of its trees and the large amount of wood that is needed to produce just a small amount of pure essential oil. Agarwood oil is by far one of the most precious essential oils in the world, with its value reported to be as high as 30,000 USD per kilogram, or up to 80,000 USD per liter. Annually, the global trade for agarwood has been estimated to be between six to eight billion USD, excluding a large number of trades that have not been recorded and accounted for [62,73]. Typically, the global economic interest in agarwood has always been focused on its pathological heavy and dense wood that is impregnated by resin. However, as mentioned earlier, agarwood only forms when affected by certain external factors, and its formation occurs infrequently and slowly in old trees. As such, the supply of agarwood from wild sources often does not meet market demand. Its rarity and immense value have further contributed to over-exploitation and indiscriminate harvesting of trees in hunting for the treasured resin, thereby leading to the rapid dwindling of wild *Aquilaria* trees. The survival of these trees in the wild is also greatly under threat as mother trees are felled and their regeneration cycle is disrupted [19,21,62,68]. Thus, the diminishing population of these trees in the wild has led to conservation efforts, such as the listing of the genus *Aquilaria* in Appendix II of the Convention of International Trade in Endangered Species of Wild Fauna and Flora (CITES), which brings its status to “potentially threatened with extinction”, as an attempt to regulate the trade of agarwood via quota restriction of goods that are exported from every country. Besides this, the International Union for Conservation of Nature and Natural Resources (IUCN) Red List of Threatened Species has also listed several *Aquilaria* species as critically endangered and vulnerable, including *A. sinensis, A. crassna*, and *A. malaccensis* [72,73,74,75].

### 3.2. Induction of Agarwood

Due to the scarcity of agarwood resources in the wild and its increasing demand, the need for producing sustainable agarwood becomes eminent, leading to the cultivation of *Aquilaria* trees in various agarwood-producing countries such as Malaysia, Thailand, China, Indonesia, Vietnam, Sri Lanka, and Australia [21]. Due to its protected status, the planting of *Aquilaria* trees is tightly regulated by the representatives of CITES in each member country. Currently, the most cultivated species of *Aquilaria* are *A. malaccensis, A. crassna*, and *A. sinensis.* Nevertheless, the frequency of natural infection remains low and is rather a matter of chance, in which it has been observed that only approximately 7 to 10% of the trees from resin in plantations. Besides this, the process of agarwood formation is also a lengthy process that could take up to 10 years. Since healthy *Aquilaria* trees do not form agarwood, which leaves them worth next to nothing, several artificial agarwood-inducing methods have also been introduced and they can be generally classified into two different groups, namely, the conventional and non-conventional methods (Table 3). The development of effective agarwood induction methods has gained considerable attention over the recent years as it is highly essential in ensuring the yield of agarwood from domesticated *Aquilaria* trees is stable. Ideally, these artificial induction methods could effectively enhance the yield of cultivated agarwood to a decent quantity for targeted downstream purposes. Therefore, the methods of artificially inducing agarwood should be practical to be employed in large-scale plantations with the aim of producing maximum supplies in the shortest possible time. In short, these artificial methods are anticipated to bring greater yields of agarwood in contrast to the natural process with a quality that resembles or is superior to that of wild agarwood [62,76,77].

The natural agarwood formation process has tremendously inspired the development of conventional induction methods. Conventional methods are generally traditional practices that have been passed down from one generation to another. For example, the usage of agarwood had a long history where multiple artificial agarwood-inducing techniques had been developed and in use for more than 1000 years in ancient China [66]. Generally, conventional techniques such as trunk breaking, wounding using a machete or ax, burning-chisel-drilling, holing, bark removal, cauterizing, and nailing have revolved around the fundamental concept of physically wounding the trees to trigger agarwood formation. Despite being cost-effective and requiring no personnel with specific scientific knowledge of agarwood, these conventional induction methods often produce agarwood with inferior quality and an uncertain yield. Moreover, agarwood is only formed at the injured area of the trees, signifying that agarwood yield directly correlates with the magnitude and number of induced physical injuries, thereby requiring more labor for the process [62,66,76,78]. On the other hand, non-conventional induction methods are designed to simulate natural events that contribute to the formation of agarwood. Briefly, these methods require a minor physical wounding to be made on the trunk as an entry point for applying a catalyst or inducer, which can be solid, semi-solid, or liquid, into the tree to trigger the formation of agarwood. Such inducers that are currently available in the market can be categorized as either chemical inducers or biological inducers [76,78].

Biological inducers refer to the application of fungi, yeast, or other natural microbial flora into *Aquilaria* trees to replicate their pathological condition in the wild. Pure culture strains that are isolated natural agarwood in a controlled environment, which can be of solid form grown on agar media in Petri dishes or liquid grown in broth media in a laboratory, are proven to be effective biological agents to induce the formation of agarwood in healthy *Aquilaria* trees [72,76,79]. Apart from that, culture strains for biological inoculum can also be “mixed”, which are mostly concocted by individual proprietors based on their aspiration in formulating inocula and experimenting with them on their own trees [76,80]. An advantage of utilizing biological inoculum is that it is eco-friendly and generally safe for handling. Nevertheless, such a technique often produces a localized and inconsistent quality of agarwood as a result of the varying fungal consortium [62]. As such, a long incubation time may be necessary prior to harvesting to produce darker wood and a better quality of agarwood. Such a long incubation period allows sufficient time for the microorganisms to multiply and maximizes colonized surface area. Subsequently, the defense mechanism of the tree will be activated by the invasion as an attempt to obstruct further foreign penetration, thereby producing agarwood resin as a barrier [78,81]. The role of fungi in artificial agarwood induction is more commonly explored as compared to bacteria. Examples of fungal isolates that have been used for artificial agarwood induction include *Melanotus flavolivens, Penicillium* spp., *Phytium* spp., *Lasiodiplodis* spp., *Botryodyplodis* spp., and *Fusarium* spp. As the outcomes of agarwood formation may differ between fungal strains, testing on a wide variety of fungal species is essential to identify the most appropriate isolate or species that can produce agarwood of high quality. However, it is important to note that such outcomes may also differ depending on the sites where they are applied [80,82,83].

Unlike biological inducers, chemical inducers are promising methods in artificially inducing agarwood as they are easy to apply, act rapidly, and are available in accurate strengths. Besides this, via the transpiration process, the time-consuming holing process can be minimized as fewer induction sites are required for the delivery of chemical inducers throughout the plant [67,84]. Therefore, such a technique is undoubtedly more appropriate for mass production with ease in quality control, which could potentially replace the methods of conventional induction and biological induction in the agarwood industry. Notably, a carefully formulated chemical inducer has been reported to produce artificial agarwood with a quality that resembles that of natural agarwood. Higher content of biologically active compounds such as sesquiterpenes has also been detected in chemically induced agarwood in contrast to mechanically wounded or biologically induced agarwood [62,85]. Initially, sodium chloride and acetic acid were used when people first attempted on chemical induction of agarwood. Upon advancement of scientific research, specialized chemicals came into the limelight as signaling molecules such as ethylene, methyl jasmonate, and salicylic acid were discovered to trigger the defense mechanisms of trees for producing agarwood. At present, commercial inducers may include content such as ferric chloride, ferrous chloride, salicylic acid, sodium methyl bisulfide, hydrogen peroxide, formic acid, cellobiose, and methyl jasmonate, which can also be added to suspension cell culture to be used in conjunction with biological inducers. Nonetheless, extensive trials must be conducted prior to their mass usage as an excessive strength of a chemical inducer may kill the tree [76,86,87].

### 3.3. Distillation of Agarwood Oil

Agarwood essential oil is the primary active ingredient of agarwood, possessing multiple pharmacological functions that are beneficial for human health [88]. As the detailed procedures of agarwood oil extraction are not within the scope of this review, we will only be providing a general overview of several techniques that have been employed for the extraction of agarwood oil. Hydro-distillation and steam distillation represent two of the most utilized techniques for the extraction of essential oil from agarwood. Briefly, in hydro-distillation, the agarwood sample is heated, either by immersing into distilled water that is brought to a boil and/or by introducing steam that is created by a separate steam boiler to it. The generated heat and/or steam breaks down the cell integrity of the plant material, leading to the release of essential oils. The essential oil molecules along with the steam travel through a pipe via a cooling tank, returning them to the liquid form, which is collected in a vat. As essential oils are typically immiscible with water, the emerging mixture of essential oil and water can be easily separated, leaving pure agarwood oil [89,90]. On the other hand, steam distillation utilizes only steam for the extraction process, in contrast to hydro-distillation which utilizes water, steam, or both for the extraction of agarwood oil [91]. Nevertheless, these conventional extraction techniques have been reported to be highly time-consuming, in which the extraction process can extend up to 16 h. Moreover, the extraction process is often incomplete with low yield efficiency while the heat is unstable to control. Other limitations of these conventional distillation methods include loss of volatile compounds, degradation of unsaturated compounds, as well as high energy consumption [92,93].

Several new methods of extraction have been developed to address the drawbacks associated with hydro-distillation and steam distillation. For example, supercritical fluid extraction (SFE) is an alternative extraction technique that is cheap, rapid, selective, and convenient. It is also found to produce high yield efficiency with a significantly greater number of fractionated compounds as compared to conventional techniques. However, as carbon dioxide represents the most desirable supercritical solvent for the extraction of essential oil, its emission during the process of SFE may be detrimental to the environment in terms of greenhouse effects [92,94]. Another method that has been developed is the accelerated solvent extraction or pressurized liquid extraction method. This technique offers the benefits of minimizing solvent consumption and allows for automated sample handling, as well as maximizing the yield of essential oil. Nevertheless, this method requires the use of organic solvents such as n-hexane which are toxic and hazardous, and it is also economically impractical when applied for the extraction of essential oil [92,93,95]. Apart from that, subcritical water extraction (SCWE) is one of the latest extraction techniques that is based on the use of water as an extractant at temperatures between 100 to 374 °C with a pressure that is sufficiently high to maintain its liquid state. Therefore, this method is known to be safe, cost-effective, and environmentally friendly. Studies have also shown that the extraction of agarwood oil using this technique resulted in a higher quantity and quality of essential oil in a shorter period as compared to hydro-distillation [92,96,97]. These suggest that SCWE may be a better alternative to existing extraction methods in terms of yield, time, and quality of extracted agarwood oil.

## 4. Potential of Agarwood Oil against Chronic Inflammatory Diseases

In recent decades, extensive studies have been conducted on agarwood in different aspects in order to identify its components, their chemical properties, biological actions, and the potential of its use as a pharmacological agent. Some of these studies aimed at extracting chemical components from the leaves, resin/oil/ or hard wood of the trees producing agarwood oil. These studies came out with a huge number of compounds many of which have proven multiple types of actions on live cells as well as on laboratory animals. The two major components found in agarwood-producing trees are sesquiterpenes and chromones among others such as aromatics, phenols, and triterpenes [98].

It is also important to note that the source, the method by which agarwood oil is formed, and the way it is analyzed make a great difference to the chemical components of the oil formed [99,100]. As it has been mentioned, due to the rarity of the trees and the prolonged time it takes for them to form (up to 4 years), traders as well as scientists worked to develop artificial ways by which they can induce the trees to form agarwood oil such as fungal inoculation and manually wounding the trees. These methods, though successful, do not produce oil with the same quality and components. The characteristic of the oil produced varied even among the different induction methods [101,102,103].

**Figure 2:**
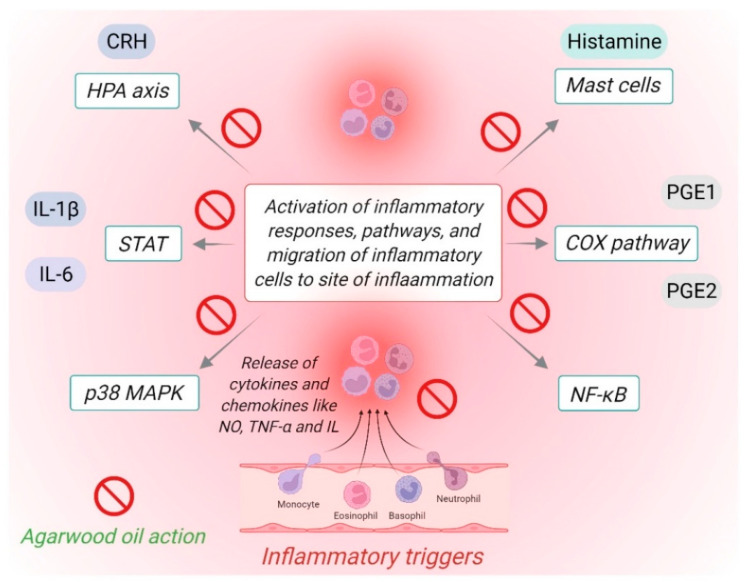
Possible mechanisms by which agarwood oil could inhibit chronic inflammatory processes.

Many of the extracted compounds were studied for their biological activities and were proven to produce many effects one of which is the anti-inflammatory property (Table 4 and Figure 2). Sesquiterpenoids are one of the most studied groups of chemicals, in one of those studies, researchers extracted multiple sesquiterpene compounds and assessed them for their anti-inflammatory activity.

In addition to studies based on certain compounds of agarwood, there has been some research done to study the effect of the oil as a whole on the processes and pathways of inflammation (Table 5). One of those studies performed an in vivo and in vitro study to evaluate the effect of agarwood oil on paw edema in mice, which were induced by carrageenan and the effect was compared with a standard treatment of 10 mg/kg of diclofenac, the result showed significant anti-inflammatory activity in a dose-dependent manner, they outlined that the approach through which this is occurring is similar to the mechanism of action of diclofenac by inhibiting the CoX enzyme and hence preventing the release of inflammatory mediators like prostaglandins [117].

A similar study was conducted by Gao et al., where they examined the effect of agarwood oil on inflammation induced in mice ears and rat paws. The authors of the study later described their results as positive for anti-inflammatory activity, which was achieved by downregulating the p-STAT3 which in turn reduced the production of the pro-inflammatory mediators IL-1β and IL-6 [118]. Peng et al. studied the chemical constituents and anti-inflammatory activity of incense smoke produced from burned agarwood. After they collected the smoke, they examined its effect on LPS-induced inflammation in RAW 264.7 cells, the result came with a strong inhibitory effect on the release of inflammatory mediators TNF-α and IL-1α [18]. These results propose that the anti-inflammatory action of agarwood is present in its smoke form suggesting the possibility of developing agarwood into an inhaled medicine that could be easily administered in respiratory conditions while avoiding the systematic effect and toxicities. Moreover, a study was conducted to identify the anti-inflammatory potential of agarwood oil on animal models using mice induced with ear inflammation and performed computerized methods (QSAR) to quantify the relationship between certain compounds identified (β-Agarofuran, α-Agarofuran, 10-epi-γ-Eudesmol, Agarospirol, Hinesol, and Jinkoh-eremol). The findings of the study came with positive anti-inflammatory action in which agarwood oil reduced the production of IL-1β, IL-6, and TNF-α. They also outlined the possibility of agarwood oil being used as a topical agent as it reduced the ear skin thickness and edema in their rat models [119].

Furthermore, agarwood oil extract from ethyl acetate was investigated to evaluate its anti-inflammatory action. Chitre et al. used rat models and induced inflammation first in their paws using carrageenan and second on their backs using cotton pellets to produce granulomas. They compared their results with rats who were given diclofenac. Finally, they found out that rats who were given the ethyl acetate extract experienced less paw edema and smaller size granulomas and this was comparable to those who were given diclofenac. In addition, they also observed fewer gastric side effects with ethyl acetate compared to diclofenac [120]. The other study was performed in human peripheral blood mononuclear cells (hPBMCs) stimulated with LPS with or without agarwood-ethyl acetate extract, they then measured the amount of TNF-α produced in both situations and found that the level was lower in samples pre-treated with agarwood. They also determined that this occurred through the selective blockage of the P38 MAPK inflammatory pathway [121].

Some researchers investigated the anti-inflammatory potential of agarwood oil on specific organs or diseases. One of the inflammatory conditions studied was intestinal injury in rats induced by 5-flurouracil intraperitoneal injections (normally used as chemotherapy for colon cancer), in the first study, they gave agarwood oil orally to the induced mice and noticed decreases in the symptoms of diarrhea, reduced weight, and low food intake. On histopathology, they found that the mice treated with agarwood had a lesser degree of damage in their intestinal mucosa and a higher level of proliferating cell nuclear antigen which promotes cell recovery. Finally, when testing the inflammatory activity, they noted a decrease in the COX-2 and TNF-α levels in the intestinal mucosal cells [122]. The second study, on the other hand, used three different types of agarwood extracted by different methods. In their results, they measured many parameters, body weight, for one, was significantly improved in the groups that were given agarwood, intestinal propulsion also showed better function compared to the group without treatment. Furthermore, they documented similar histopathological results with decreased mucosal damage. When measuring the inflammatory mediators, they recorded a decrease in the levels of NO, IL-17, IL-33, and increased IL-10, which acts as an inhibitor of the inflammatory process. There was also an increase in the glutathione and superoxide dismutase activity and inhibition of the NF-κB inflammatory pathway [123]. The results of both these studies showed a strong indication of the effect of agarwood on the inflammation of the intestinal mucosa when given orally through multiple pathways.

The same previous team performed a similar study but on gastric inflammation where they introduced the agarwood orally to the mice after which they included gastric ulcers using pure ethanol. The results came with a strong protective effect on the gastric mucosa and a decrease in the signs of inflammation in the area including swelling, and inflammatory cells recruitment in a dose-dependent manner [124]. Moreover, the aforementioned study by Chitre et al. also spotted the protective benefit of agarwood oil on the gastric mucosa compared to diclofenac in preventing the occurrence of gastric ulcers, which further supports the claim of agarwood use to prevent inflammation in the gastric mucosa [120].

Rahman et al. provided another proof in an in vitro and in vivo study to investigate the possibility of using agarwood oil in treating arthritis, in their study, they used the BSA method in their in vitro experiment in which it showed a reduction in the heat-induced protein denaturation that is thought to be one of the mechanisms leading to the development of arthritis. on the other hand, in the treatment of Freund’s-adjuvant-induced arthritic rat models with agarwood, they recorded a reduction in the development of paw edema, they also noted a significant improvement in the blood parameters of rats treated with agarwood, and finally, on radiographic examination, they noticed a significant reduction in the swelling and joint widening that represents arthritis and joints returned to near normal condition after treatment with agarwood [125]. Further investigations need to be conducted after these promising results to understand the molecular mechanism of agarwood oil in arthritis. This would bring hope to patients suffering from the disease and from the side effects of its medications.

Another observation was made by Hamouda et al. where they had observed the anti-inflammatory property of agarwood on rats induced with inflammation in the liver or in the brain induced by methanol injection. They evaluated their results based on multiple inflammatory pathways and all lead to strong inhibitory action that brought back the NO, MDA, ACHE, COX-2, LOX, TNF-α, Caspase-3, MAO, and DNAF proinflammatory mediators and neurotransmitters to near normal levels [126]. In the same area, other studies were done to prove the action of agarwood on stress-induced depression and anxiety. One study used in vivo stressed rats and performed a number of behavioral tests after giving them agarwood, and they found a strong antidepressant and anxiolytic effect of agarwood which they believed was due to inhibition and down regulation of a number of cytokines (IL-1α, IL-1β, and IL-6) that activate the HPA axis and eventually lead to depression and anxiety [127]. The second study used similar methods on rats to perform their experiments, which revealed strong anti-depressant and anti-stress effects that they observed by finding reduced levels of lipid peroxidation, NO, TNF-α, IL-1β, cortisol, COX-2, LOX, AST, ALT, and lipids, which they believed is mainly due to suppression of these cytokines following the suppression of cortisol which promoted their secretion in the first place [128]. According to recent studies, the neuroimmune-endocrine axis plays an important role in the pathophysiology of both depression and anxiety. After stressful triggers in the brain, a number of inflammatory cytokines are released including IL-1β, IL-6, and TNF-α, these, in turn, affect the action of neurotransmitters and hormonal balance in the HPA axis in the brain, hence disturbing emotional balance and producing symptoms of depression and anxiety. Additionally, when the stressor affecting the brain and inducing inflammation stays for prolonged periods, this induces neuronal damage and activates further inflammatory pathways and mediators like NO, PGE, and COX [129]. Thus, agarwood oil is a good potential candidate to develop a newer agent that can act on both diseases while avoiding the side effects of conventional treatments.

Even though there is not much research done to study the effect of agarwood oil on respiratory cells or tissue per se, the aforementioned studies propose that agarwood oil can be involved in targeting certain inflammatory pathways in the lungs. NF-κB and p38 MAPK pathways are important in the pathophysiology of asthma and COPD. NF-κB is activated by inflammatory mediators like IL-1 and TNF-α, cigarette smoking, viruses, inflammatory cells, and other pollutants [130,131]. p38 MAPK pathway, on the other hand, is a potential therapeutic target for inflammatory diseases including COPD [132]. Apart from that, Inoue et al. highlighted the role of agarwood oil in blocking histamine release from mast cells in rats, a finding that strongly encourages the use of agarwood oil in all allergy-related diseases including asthma [133].

## 5. Conclusions and Future Perspectives

The information from the scientific literature provided in our review clearly suggests that agarwood oil possesses potent anti-inflammatory properties with promising prospects in developing drugs. The versatile nature of agarwood-based formulations works in different routes of administration, which can be utilized to target multiple inflammatory disorders such as asthma and COPD at an early stage, or prevent exacerbations, and even relieve symptoms of exacerbations. The promising anti-inflammatory activity of agarwood is due to its potency to inhibit the secretion/production/expression of a range of inflammatory mediators such as NO, TNF-α, IL-6, IL-1β, and PGE2 that are involved in the pathogenesis of a majority of inflammatory diseases. As such, agarwood could be a noble “drug candidate” for pharmaceuticals and “cosmetic candidate” for cosmeceutical companies. Although many studies have proven the effectiveness of agarwood oil on multiple disorders, these studies require further investigations and evaluation, especially with regard to the toxicity and adverse effects of agarwood. There have not been enough data to determine all the types of toxicities of agarwood oil or the degree of toxicity and the exact safe dose on different kinds of cells. The correlation of in vitro findings on agarwood with in vivo data is essential to further validate its therapeutic potential and progress into the clinical use of agarwood in the form of various dosage and administration routes. Further extensive studies need to be conducted on the possible methods by which agarwood oil can be developed into a clinical drug, its effective doses, toxicities, and the validity of the mentioned pathways to act as an inhibitor to respiratory inflammation [134]. In summary, agarwood oil has the potential to make a great impact on the future management of many chronic inflammatory diseases by blocking some of the major pathways by which inflammation occurs including NF-κB and p38 MAPK.

## Figures and Tables

**Table 1:** Summary of the inflammatory processes in different body organs.

Place of Inflammation	Causes	Mediators	Consequences	Reference
Intestine	Infections	Campylobacter and Salmonella	Inflammatory bowel disease (IBD)	[32]
	Food	Food with fatty acid compounds	Crohn’s disease	[33]
	Dysbiosis	Gut microbiome	Acute gastroenteritis, IBD	[34]
	Environmental factors	Smoking, nutrition, climate, pollution	IBD	[35]
Stomach lining	Infections	*H. pylori*	Ulcerative colitis	[36]
	NSAIDs	Reduced prostaglandin production due to inhibition of COX1 and COX2	Colitis, IBD	[37]
	Psychological stress,	Increased acid load, effects of hypothalamic-pituitary-adrenal axis activation on healing, altered blood flow, or cytokine-mediated impairment of mucosal defenses	Peptic ulcer	[38]
	Physical stress like brain injury	Traumatic head injury can cause increased intracranial pressure and lead to overstimulation of the vagus nerve and increased secretion of gastric acid.	Cushing’s ulcer.	[39]
Joint	Hyperuricemia	Increase uric acid deposition in joint	Gout (Joint inflammation)	[40]
	Genetics	HLA-DRB1 alleles: HLA-DRB1*04, HLA-DRB1*01, and HLA-DRB1*10.	Rheumatoid arthritis	[40,41]
		Mutations in genes encoding types II, IV, V, and VI collagens		
	Environmental/Diet factors	Smoking and alcohol intake	Rheumatoid arthritis	[42]
	Autoimmune	Anti-citrullinated protein/peptide antibodies	Rheumatoid arthritis	[42]
Brain	Infections	Herpes simplex	Encephalitis	[43,44]
		Human immune deficiency virus		
	Autoimmune disorder	Anti-N-methyl-D-aspartate receptor (anti-NMDA) encephalitis	Autoimmune encephalitis	[45,46]
			Autoimmune Meningitis	
	Ischemia	Blocking or narrowing of artery leading to brain	Vascular brain injury, Stroke	[47]
Lung	Cigarette smoke	Components of cigarette smoke that mediate oxidative stress and inflammatory	Airway inflammation, COPD	[48]
	Allergen	Increase inflammatory cytokines by allergens such as Ovalbumin	Airway inflammation, allergic asthma	[49]
	Air pollution	Particulate matter (PM) from traffic, industries, and ozone	Airway disease	[50]
	Infections	Influenza-induced exacerbation	Airway inflammation, Chronic lung disease	[51]
	Dysbiosis	Lung microbiome	Airway inflammation, Chronic lung disease	[52]
	Bushfire/Wildfire smoke	Complex mix of inspirable particles, volatile organics, aldehydes, carbon monoxide, and particulate matter (PM)	Airway inflammation, Chronic lung disease	[53]

**Table 2:** Various species of *Aquilaria* and their distribution.

Species	Agarwood-Producing	Distribution	Reference
*Aquilaria acuminata*	Yes	Thailand, Indonesia, Papua New Guinea, Philippines	[69]
*Aquilaria apiculata*	Yes	Philippines	[68,69]
*Aquilaria baillonii*	Yes	Cambodia, Laos, Thailand	[68,69]
*Aquilaria banaensis*	Yes	Vietnam	[68,69]
*Aquilaria beccariana*	Yes	Malaysia, Indonesia, Brunei	[68,69]
*Aquilaria brachyantha*	Yes	Malaysia, Philippines	[68,69]
*Aquilaria citrincarpa*	No	Philippines	[68,69]
*Aquilaria crassna*	Yes	Thailand, Vietnam, Laos, India, Cambodia, Malaysia	[68,69]
*Aquilaria cumingiana*	Yes	Indonesia, Philippines	[68,69]
*Aquilaria filaria*	Yes	Indonesia, Singapore, Malaysia, China, Philippines	[68,69]
*Aquilaria grandiflora*	Yes	China	[69]
*Aquilaria hirta*	Yes	Indonesia, Malaysia, Thailand, Singapore	[68,69]
*Aquilaria khasiana*	Yes	Bangladesh, India	[68,69]
*Aquilaria malaccensis*	Yes	Bhutan, Thailand, Malaysia, India, Vietnam, Bangladesh, Indonesia, Iran, Myanmar, Singapore, Philippines	[68,70]
*Aquilaria microcarpa*	Yes	Indonesia, Malaysia, Singapore	[68]
*Aquilaria ophispermum*	No	Indonesia	[68]
*Aquilaria parvifolia*	No	Philippines	[68]
*Aquilaria pentandra*	No	Bhutan, Laos, Thailand, Myanmar	[68]
*Aquilaria rostrata*	Yes	Malaysia	[68]
*Aquilaria rugosa*	No	Thailand, Vietnam	[68]
*Aquilaria sinensis*	Yes	China	[68,71]
*Aquilaria subintegra*	Yes	Malaysia, Thailand	[68]
*Aquilaria urdanetensis*	No	Philippines	[68]
*Aquilaria yunnanensis*	No	China	[68]

**Table 3:** Summary of various agarwood-inducing methods.

Type	Examples	Concept	Advantage	Disadvantage	Reference
Natural	Thunder strike	Wounds are created which then triggers the activation of the tree’s defense system, thereby producing resin	High-quality agarwood Does not require cultivation, plantation, and artificial induction No cost required and eco-friendly	Extremely low agarwood yield Unsustainable and undetermined where agarwood formation is a matter of chance Requires extremely long duration to produce high-quality agarwood Requires extensive and indiscriminate harvesting of wild trees	[73,76]
	Animal grazing				
	Pest and disease				
	Broken branches				
	Microbial invasion				
Artificial conventional	Physical wounding	Mimics natural factors by creating physical wounds on the trees which will then trigger the formation of agarwood via tree’s defense mechanism	Cost-effective Does not require personnel with specific knowledge in agarwood	Laborious Localized formation of agarwood only at the wounded area Agarwood formation correlates with the magnitude of induced injury Inferior quality of agarwood with an uncertain yield	[76]
	Cauterizing				
	Nailing				
	Holing				
	Bark removal				
	Trunk pruning				
	Burning-chisel-drilling				
Artificial biological	Fungal strains such as *Melanotus flavolivens*, *Penicillium* spp., *Phytium* spp., *Lasiodiplodis* spp., *Botryodyplodis* spp., and *Fusarium* spp.	Introduction of microbial cultures into *Aquilaria* trees to mimic its pathological infection, thereby triggering the tree’s defense mechanism	Eco-friendly and safe for handling Microbial cultures can be prepared at a low cost and are readily available	Long incubation time is required to produce high-quality agarwood	[73]
				Time-consuming holing process for inoculating microbial cultures	
				Inconsistency in agarwood quality depending on fungal species and site of inoculation	
Artificial chemical	Chemicals or signaling molecules such as ferric chloride, ferrous chloride, salicylic acid, sodium methyl bisulfide, hydrogen peroxide, formic acid, cellobiose, and methyl jasmonate	Direct induction of tree’s defense mechanism for the secretion of resin	Easy to apply with rapid action	An appropriate amount must be applied as an excess could kill the tree Skeptical impact on the environment and human health	[73]
			Minimize the time required for holing processes		
			Suitable for large scale plantations		
			Ease of quality control		
			High-quality agarwood with high and consistent yields		
			Agarwood formation can be induced in the whole tree		

**Table 4:** Summary of studies on compounds extracted from agarwood with proven anti-inflammatory action.

Compound	Study Model	Anti-Inflammatory Outcomes		Reference
		Inflammatory Pathways	Key Findings	
2-(2-phenylethyl) chromone	In vitro study on RAW 264.7 cells.	Inhibit the activation of MAPK and STAT pathways.	Inhibit the production of NO, TNF-α, IL-6, IL-1β, PGE2.	[104]
	In vitro study on RAW 264.7 cells.	Inhibit NF-κB activation.	Inhibit the production of NO.	[105]
	In vitro study on RAW 264.7 cells.	Not specified.	Inhibit the production of NO.	[106,107,108,109,110,111,112]
Sesquiterpenoids	In vitro study on RAW 264.7 cells.	Not specified.	Inhibit the production of NO.	[17]
	In vitro study on RAW 264.7 cells.	Not specified.	Inhibit the production of NO.	[113]
	In vitro study on RAW 264.7 cells.	Not specified.	Inhibit the production of NO.	[114]
Others: β-caryophyllene	In vivo study on rats with paw edema induced with carrageenan.	Not specified.	Reduced edema in rat paws.	[115]
α-humulene	Ovalbumin induced mice model of allergic asthma	inhibition of the activation of p65 NF-kB and c-Jun AP-1	reduction of eosinophils in the bronchoalveolar lavage fluid as well as inflammatory mediators such as IFN-γ, IL-5, CCL11, and LTB4 levels. Decrease in the production of IL-5 in the mediastinal lymph nodes, mucus secretions in the lungs.	[116]

**Table 5:** Summary of studies proving the anti-inflammatory properties of agarwood oil.

Study Model(s)	Concentration	Study Duration	Anti-Inflammatory Outcomes		Reference
			Inflammatory Pathways	Key Findings	
In vivo and in vitro study on carrageenan-induced rat paw edema and HRBC stabilization method	In vivo: 50 and 100 mg/kg	In vivo: 4 h	Inhibition of the cyclooxygenase (COX) inflammatory pathway	Strong inhibition of rat paw edema. Inhibition of the release of prostaglandins. HRBC membrane stabilization.	[117]
	In vitro: 100, 250, and 500 mcg/mL		Inhibition of cell membrane lysis induced by hypotonicity.		
In vivo study on carrageenan-induced rat paw edema and xylene-induced ear edema in mice	Mice: 60 to 960 mg/kg	Not specified	Inhibit the expression p-STAT3 gene.	Reduce the production of IL-1β and IL-6.	[118]
	Rats: 680 mg/kg				
In vitro study on RAW 264.7 cells	Not specified	Not specified	Not specified.	Inhibit the release of TNF-α and IL-1α.	[18]
In vivo study on mice induced with ear inflammation and in silico studies: ADME and QSAR	In vivo: 20 uL/ear for 3 times	24 h	Not specified.	Reduce inflammation in mice ears.	[119]
				Inhibit the release of IL-1β, IL-6, and TNF-α.	
				ADME and QSAR results corresponding to anti-inflammatory activity.	
In vivo study on rats with paw edema induced with carrageenan and with granuloma induced with cotton pellets	50, 100 and 200 mg/kg	Carrageenan-induced paw edema: 3 h Cotton pellets-induced granuloma: 7 days	Not specified.	Inhibit the activity of prostaglandins (PGE2 and PGI2).	[120]
				Reduced edema in rat paws.	
				Smaller size granuloma compared to control group.	
In vitro study on hPBMCs	0.5, 1.0, 1.5, 2.0, 2.5, and 3.0 mg/mL	24 h	Inhibit the p38 MAPK activation.	Inhibit the production of TNF-α.	[121]
In vivo study on mice with intestinal injury induced by 5-flurouracil	200, 400, and 800 mg/kg	7 days	Inhibiting the oxidative stress.	Less symptoms of intestinal inflammation.	[122]
			Inhibiting the expression of inflammatory mediators.	Less tissue inflammation observed on histopathology and improved recovery.	
			Inhibiting the NF-κB pathway.	Decreased levels of COX-2 and TNF-α inflammatory mediators in the intestinal cells.	
In vivo study on mice with intestinal injury induced by 5-flurouracil	0.71, 1.42 and 2.84 g/kg	14 days	Inhibiting oxidative stress. Inhibiting the mRNA expression of inflammatory pathways and mediators.	Improved body weight and intestinal propulsion.	[123]
				Less mucosal injury.	
				Decreased levels of NO and increased glutathione and superoxide dismutase activity.	
				Decreased the levels of IL-17, IL-33, and increased IL-10.	
				Inhibiting the NF-κB pathway	
In vivo study on mice with gastric ulcers induced by ethanol	0.71, 1.42 and 2.84 g/kg	7 days	Inhibiting oxidative stress. Inhibiting the mRNA expression of inflammatory pathways and mediators.	Protective effect against gastric ulcer and lesser degree of inflammation.	[124]
				Decreased levels of IL-1β, IL-6, and increased level of IL-10.	
				Inhibition of the NF-κB and p38 MAPK pathways.	
In vitro bovine serum protein (BSA) denaturation method and in vivo Freund’s-adjuvant-induced arthritic rat model	In vivo: 125 and 250 mg/kg	In vivo: 21 days	Inhibition of protein denaturation.	Reduced paw edema by gross observation and radiography.	[125]
	In vitro: 100, 250 and 500 mcg/mL		Inhibition of inflammatory mediators.	Improved hematological parameters.	
In vivo study on methanol induced inflammation in livers and brains of rats	100 mg/kg	35 days	Inhibit oxidative stress and apoptosis.	Inhibit the release of NO, MDA, ACHE, COX-2, LOX, TNF-α, Caspase-3, MAO, and DNAF neurotransmitters and pro-inflammatory mediators.	[126]
In vivo study on stress-induced anxiety and depression in rats	10, 20 and 40 mg/kg	10 days	Decreases the levels of IL-1α, IL-1β, and IL-6 in serum. Downregulated the iNOS in the cerebral cortex and hippocampus.	Antidepressant effect.	[127]
				Anxiolytic effect.	
				Decreased levels of ACTH and CORT serum.	
In vivo study on rats with stress-induced with epinephrine	100 mg/kg	21 days	Inhibition of cortisol production.	Reduced levels of lipid peroxidation, NO, TNF-α, IL-1β, cortisol, COX-2, LOX, AST, ALT, and lipids.	[128]

## Data Availability

Not applicable.
